# Ocean Species Discoveries 28–30 — new species of chitons (Mollusca, Polyplacophora) and a public naming competition

**DOI:** 10.3897/BDJ.14.e180491

**Published:** 2026-02-06

**Authors:** Senckenberg Ocean Species Alliance (SOSA), Chong Chen, Hosea Frank, Laura Kraniotis, Yumi Nakadera, Enrico Schwabe, Julia D. Sigwart, Bianca Trautwein, Katarzyna Vončina

**Affiliations:** 1 Senckenberg Research Institute and Museum, Frankfurt am Main, Germany Senckenberg Research Institute and Museum Frankfurt am Main Germany; 2 X-STAR, Japan Agency for Marine-Earth Science and Technology (JAMSTEC), Yokosuka, Kanagawa, Japan X-STAR, Japan Agency for Marine-Earth Science and Technology (JAMSTEC) Yokosuka, Kanagawa Japan; 3 Independent researcher, New Jersey, United States of America Independent researcher New Jersey United States of America; 4 Senckenberg Society for Nature Research, Frankfurt, Germany Senckenberg Society for Nature Research Frankfurt Germany; 5 Bavarian State Collection for Zoology, München, Germany Bavarian State Collection for Zoology München Germany; 6 Institute of Ecology, Diversity and Evolution, Faculty of Biological Sciences, Goethe University Frankfurt, Frankfurt am Main, Germany Institute of Ecology, Diversity and Evolution, Faculty of Biological Sciences, Goethe University Frankfurt Frankfurt am Main Germany

**Keywords:** Ferreiraellidae, Acanthochitonidae, Mopaliidae, sunken wood, taxonomy, True Facts, YouTube

## Abstract

**Background:**

Three new chitons are reported from distinct and underexplored marine habitats: *Ferreiraella populi* sp. nov. from deep-sea sunken wood off Japan in the western Pacific, *Notoplax madagascariensis* sp. nov. from Madagascar, and the carnivorous *Placiphorella granulosa* sp. nov. from Papua New Guinea, occurring from the sublittoral into the bathyal zone. These findings broaden current understanding of chiton diversity and emphasise how much remains undocumented in the deep-sea and tropical systems. The discovery of *Notoplax madagascariensis* sp. nov. is particularly relevant for ongoing efforts to resolve generic limits within Cryptoplacoidea, where both taxonomic and molecular evidence remain incomplete.

**New information:**

The three species described here add substantially to the known diversity and ecological breadth of chitons. The species epithet of the new *Ferreiraella* species was the outcome of a public naming competition, an initiative intended to raise awareness of taxonomy and enhance public engagement with species discovery. All three chitons illustrate the value of targeted sampling across poorly-studied habitats.

## Introduction

The “Ocean Species Discoveries” series of papers have presented taxonomic contributions of new species and genera across marine invertebrate phyla (Senckenberg Ocean Species Alliance (SOSA) et al. 2024, Senckenberg Ocean Species Alliance (SOSA) et al. 2025). The series was devised to enable streamlined publication of stand-alone species descriptions for marine invertebrate species, with a collection of contributions from many taxonomists. Authorship of the article includes the contributors to the collaborative taxonomic process, while the species authority remains with the contributing specialist. Taxonomy is an increasingly collaborative discipline, although the rate of change lags behind other disciplines (Sigwart et al. 2023).

Chitons (class Polyplacophora) are a distinctive group of marine molluscs characterised by their eight articulating shell plates and dorsoventrally flattened bodies (Schwabe 2010). They inhabit a wide range of marine environments, from intertidal zones to deep-sea habitats, with the highest diversity in tropical regions. Over 1000 living species have been described to date and new taxa are discovered annually. However, much of their biodiversity likely remains undocumented, particularly in regions that remain underexplored for chitons, such as Melanesia and the Coral Triangle (Schwabe 2006), areas known for their exceptionally high marine diversity. Deep-sea chitons also remain insufficiently studied (Schwabe 2008).

The genus *Ferreiraella* Sirenko, 1988 contains 10 species including this new species, and two recognised subspecies of *F. xylophaga* (Sirenko 1988, Sigwart and Sirenko 2012, Senckenberg Ocean Species Alliance (SOSA) et al. 2025). The genus, the only member of the family Ferreiraellidae, is endemic to sunken wood. The species reported here was included in an earlier publication on the discovery of a novel community of a late successional stage on sunken wood, found in the western Pacific near Japan (Chen et al. 2024) and is here formally described from material collected in a second expedition to the same site.

The genus *Placiphorella* Dall, 1879 (Dall 1879) represents one of the three chiton lineages that have independently evolved an ambush-predatory lifestyle (Saito and Okutani 1992). Its species occur from intertidal to deep-sea habitats, including methane seeps (*P. okutanii* Saito, Fujikura & Tsuchida, 2008; *Placiphorella methanophila* Vončina, 2024). Members of the genus are distinguished by extended anterior girdle bearing scaled bristles and short, broad valves forming a nearly circular body. The anterior flap, equipped with precephalic tentacles, can rapidly close to capture small invertebrates such as worms and crustaceans (McLean 1962). Fifteen *Placiphorella* species are mainly distributed across the northern Pacific, with additional records from New Zealand and a single record from Indonesia (*P. albitestae* Is. Taki, 1954; Kaas (1985)), but none from Melanesia. Few studies have treated the chiton fauna of Papua New Guinea (Ashby 1923, Leloup 1981a, Schwabe 2006, Sirenko 2020) and none of them reported *Placiphorella*. The most comprehensive revisions of the genus remain those of Clark (1994) and Kaas and Van Belle (1994).

The genus *Notoplax* Adams, 1862 (Adams 1862) includes medium- to large-sized chitons characterised by a moderately to strongly reduced tegmentum, a broad fleshy girdle that often encroaches deeply between the valves and asymmetrical radula rows (Gowlett-Holmes 1991, Saito 2004, Sirenko and Saito 2017). Forty-two species are currently recognised, many of which have been found associated with sponges (Kaas et al. 1998, Kangas and Shepherd 2013). The genus is widely distributed across the Indo-Pacific, with greatest diversity from Japan southward to New Zealand. Reports from the western Indian Ocean, including Madagascar, are scarce. Leloup (1981b) recorded an *Acanthochiton* sp., subsequently recognised as a record of *Notoplax* by Kaas (1986). Other records from the region refer only to *Leptoplax curvisetosa* (previously classified within *Notoplax*) in living and fossil records (Kaas 1986, Dell’Angelo et al. 2004).

The present study describes and illustrates three new species of chitons: *Notoplax madagascariensis* sp. nov. from the sublittoral zone off Madagascar, and *Placiphorella granulosa* sp. nov. from the bathyal zone off Papua New Guinea. For the new *Ferreiraella* species described herein, we offered an open public competition to suggest names, requesting creative responses to information provided via social media about this species and the taxonomic description process. The authors selected a name based on the public suggestions; the decision process included consideration of the enthusiasm for some suggestions among the community of people who engaged in the process. The final selection conforms to the requirements of the International Code for Zoological Nomenclature.

## Materials and methods

Material examined in this study was collected during the French expeditions MIRIKY (2009), MAINBAZA (2009) and BIOPAPUA (2010), and a Japanese expedition by Japan Agency for Marine-Earth Science and Technology (JAMSTEC) using the Deep Submergence Vehicle (DSV) *Shinkai 6500* (May 2024). The systematic classification follows Sirenko (2006) with slight modifications, the morphological terminology follows Schwabe (2010).

Whole specimens were photographed using a Canon EOS 6D camera equipped with an EF 100 mm 1:2.8 IS USM macro lens, and serial images were stacked using Helicon Focus (v. 5.3 X64) software. The images of dissected valves were taken using a motorised Nikon SMZ25 stereomicroscope with an attached Nikon Digital Sight 10 camera. To create stacked images, Nikon NIS Elements Basic Research (v. 5.42.04) software was used.

For scanning electron microscopy (SEM), the anterior valves (I-IV), tail valve, and radulae were dissected, cleaned in a diluted bleach solution (1:1 with H_2_O) and rinsed thoroughly with distilled water. Small fragments of the girdle were removed, treated wih dilute bleach as above, and air dried. All samples were placed on SEM stubs using double-sided adhesive tabs, gold-sputtered with CamScan CS 24 (Cambridge Instruments) at the Senckenberg Research Institute, Frankfurt, and examined with a Hitachi TM4000 tabletop SEM.

For DNA barcoding, a small fragment of tissue from the foot was used for DNA extraction with the QIAamp DNA Micro Kit (QIAGEN), following the manufacturer’s protocol. The mitochondrial cytochrome c oxidase subunit I (COI) gene was amplified using the primers LCO1490 and HCO2198 (Folmer et al. 1994) and TaKaRa Taq HS Perfect Mix (TaKaRa). PCR conditions were as follows: an initial denaturation step at 94ºC for 5 minutes; then 35 cycles of denaturation at 94ºC for 45 seconds, annealing at 50ºC for 45 seconds and extension at 72°C for 1 minute and 30 seconds; with a final extension at 72°C for 5 minutes. The COI sequences from the *Placiphorella* holotype (MNHN-IM-2019-34864) and *Ferreiraella* holotype (SMF 383139) are deposited in GenBank under accession numbers [to be added]. DNA amplification of *Notoplax* was unsuccessful. However, genomic data obtained through targeted enrichment of ultraconserved elements (UCEs) are deposited in NCBI under accession numbers SAMN51331557 (MNHN-IM-2007-38001) and SAMN51331540 (SMF 383079) (Vončina et al., unpublished data).

### Abbreviations

Terminology in these descriptions, including the indications of size and morphological proportions, follows a standardised approach for chitons documented by Schwabe (2010).

Abbreviations: BL – body length; L – length; W – width; pms – post-mucronal slope; MNHN - Muséum national d'Histoire naturelle, Paris, France; SMF – Senckenberg Research Institute and Natural History Museum Frankfurt, Frankfurt, Germany; NMST – National Museum of Nature and Science, Japan.

## Data resources

A 3D model of *Ferreiraella populi* sp. nov., suitable for printing, is available for download from MorphoSource. Download URL: https://www.morphosource.org/concern/media/000810929.

## Taxon treatments

### Ferreiraella populi

Sigwart sp. nov.

920FA688-92F7-54DC-AABD-87745EB11E37

00C12EDF-5D9E-4451-B590-B0BFD8B5D7F6

Ferreiraella sp. Chen et al. 2024: figs. 2a, 2b, 2d and 3v.

#### Materials

- **Type status:** Holotype. **Occurrence:** catalogNumber: SMF 383139; recordedBy: Chong Chen; individualCount: 1; lifeStage: adult; occurrenceID: A8BDC46D-A6E3-5EF8-82D8-57663382AEA4; **Taxon:** scientificName: Ferrieraella populi; kingdom: Animalia; phylum: Mollusca; class: Polyplacophora; order: Lepidopleurida; family:  Ferreiraellidae; genus:  Ferreiraella; specificEpithet: populi; taxonRank: species; scientificNameAuthorship: Sigwart; nomenclaturalCode: ICZN; **Location:** higherGeography: Izu-Ogasawara Trench; country: Japan; verbatimLocality: Izu-Ogasawara Trench, West of KEO; verbatimDepth: 5506; minimumDepthInMeters: 5506; maximumDepthInMeters: 5506; verbatimCoordinates: 32°34.6760' N, 143°46.1235' E; verbatimLatitude: 32°34.6760' N; verbatimLongitude: 143°46.1235' E; decimalLatitude: 32.577933; decimalLongitude: 143.768725; **Identification:** identifiedBy: Juila Sigwart; dateIdentified: 2024; **Event:** samplingProtocol: suction sampler; eventDate: 2024-05; year: 2024; month: 5; habitat: woodfall; **Record Level:** language: en; institutionID: 16946ec1-8db3-45b8-b084-7644384cc5f5; basisOfRecord: PreservedSpecimen; **Material Entity:** associatedSequences: https://www.ncbi.nlm.nih.gov/nuccore/PX804841
- **Type status:** Paratype. **Occurrence:** catalogNumber: SMF 383138; recordedBy: Chong Chen; individualCount: 1; lifeStage: adult; occurrenceID: 0567F91E-526E-5EC1-BBE8-BAC13E40468D; **Taxon:** scientificName: Ferrieraella populi; kingdom: Animalia; phylum: Mollusca; class: Polyplacophora; order: Lepidopleurida; family:  Ferreiraellidae; genus:  Ferreiraella; specificEpithet: populi; taxonRank: species; scientificNameAuthorship: Sigwart; nomenclaturalCode: ICZN; **Location:** higherGeography: Izu-Ogasawara Trench; country: Japan; verbatimLocality: Izu-Ogasawara Trench, West of KEO; verbatimDepth: 5506; minimumDepthInMeters: 5506; maximumDepthInMeters: 5506; verbatimCoordinates: 32°34.6760' N, 143°46.1235' E; verbatimLatitude: 32°34.6760' N; verbatimLongitude: 143°46.1235' E; decimalLatitude: 32.577933; decimalLongitude: 143.768725; **Identification:** identifiedBy: Juila Sigwart; dateIdentified: 2024; **Event:** samplingProtocol: suction sampler; eventDate: 2024-05; year: 2024; month: 5; habitat: woodfall; **Record Level:** language: en; institutionID: 16946ec1-8db3-45b8-b084-7644384cc5f5; basisOfRecord: PreservedSpecimen
- **Type status:** Paratype. **Occurrence:** catalogNumber: SMF 383140; recordedBy: Chong Chen; individualCount: 1; lifeStage: adult; occurrenceID: 386AD89A-1A96-5946-A45C-0CB0E195EA10; **Taxon:** scientificName: Ferrieraella populi; kingdom: Animalia; phylum: Mollusca; class: Polyplacophora; order: Lepidopleurida; family:  Ferreiraellidae; genus:  Ferreiraella; specificEpithet: populi; taxonRank: species; scientificNameAuthorship: Sigwart; nomenclaturalCode: ICZN; **Location:** higherGeography: Izu-Ogasawara Trench; country: Japan; verbatimLocality: Izu-Ogasawara Trench, West of KEO; verbatimDepth: 5506; minimumDepthInMeters: 5506; maximumDepthInMeters: 5506; verbatimCoordinates: 32°34.6760' N, 143°46.1235' E; verbatimLatitude: 32°34.6760' N; verbatimLongitude: 143°46.1235' E; decimalLatitude: 32.577933; decimalLongitude: 143.768725; **Identification:** identifiedBy: Juila Sigwart; dateIdentified: 2024; **Event:** samplingProtocol: suction sampler; eventDate: 2024-05; year: 2024; month: 5; habitat: woodfall; **Record Level:** language: en; institutionID: 16946ec1-8db3-45b8-b084-7644384cc5f5; basisOfRecord: PreservedSpecimen
- **Type status:** Paratype. **Occurrence:** catalogNumber: SMF 383141; recordedBy: Chong Chen; individualCount: 1; lifeStage: adult; occurrenceID: 752C0E55-09AE-5399-9EF4-83381C60D4A4; **Taxon:** scientificName: Ferrieraella populi; kingdom: Animalia; phylum: Mollusca; class: Polyplacophora; order: Lepidopleurida; family:  Ferreiraellidae; genus:  Ferreiraella; specificEpithet: populi; taxonRank: species; scientificNameAuthorship: Sigwart; nomenclaturalCode: ICZN; **Location:** higherGeography: Izu-Ogasawara Trench; country: Japan; verbatimLocality: Izu-Ogasawara Trench, West of KEO; verbatimDepth: 5506; minimumDepthInMeters: 5506; maximumDepthInMeters: 5506; verbatimCoordinates: 32°34.6760' N, 143°46.1235' E; verbatimLatitude: 32°34.6760' N; verbatimLongitude: 143°46.1235' E; decimalLatitude: 32.577933; decimalLongitude: 143.768725; **Identification:** identifiedBy: Juila Sigwart; dateIdentified: 2024; **Event:** samplingProtocol: suction sampler; eventDate: 2024-05; year: 2024; month: 5; habitat: woodfall; **Record Level:** language: en; institutionID: 16946ec1-8db3-45b8-b084-7644384cc5f5; basisOfRecord: PreservedSpecimen
- **Type status:** Paratype. **Occurrence:** catalogNumber: SMF 383142; recordedBy: Chong Chen; individualCount: 1; lifeStage: adult; occurrenceID: 8B3FC026-68C4-580F-ABC1-34463B13C036; **Taxon:** scientificName: Ferrieraella populi; kingdom: Animalia; phylum: Mollusca; class: Polyplacophora; order: Lepidopleurida; family:  Ferreiraellidae; genus:  Ferreiraella; specificEpithet: populi; taxonRank: species; scientificNameAuthorship: Sigwart; nomenclaturalCode: ICZN; **Location:** higherGeography: Izu-Ogasawara Trench; country: Japan; verbatimLocality: Izu-Ogasawara Trench, West of KEO; verbatimDepth: 5506; minimumDepthInMeters: 5506; maximumDepthInMeters: 5506; verbatimCoordinates: 32°34.6760' N, 143°46.1235' E; verbatimLatitude: 32°34.6760' N; verbatimLongitude: 143°46.1235' E; decimalLatitude: 32.577933; decimalLongitude: 143.768725; **Identification:** identifiedBy: Juila Sigwart; dateIdentified: 2024; **Event:** samplingProtocol: suction sampler; eventDate: 2024-05; year: 2024; month: 5; habitat: woodfall; **Record Level:** language: en; institutionID: 16946ec1-8db3-45b8-b084-7644384cc5f5; basisOfRecord: PreservedSpecimen
- **Type status:** Paratype. **Occurrence:** catalogNumber: SMF 383143; recordedBy: Chong Chen; individualCount: 1; lifeStage: adult; occurrenceID: 830CF36E-7701-5652-98FD-764093FFFD0C; **Taxon:** scientificName: Ferrieraella populi; kingdom: Animalia; phylum: Mollusca; class: Polyplacophora; order: Lepidopleurida; family:  Ferreiraellidae; genus:  Ferreiraella; specificEpithet: populi; taxonRank: species; scientificNameAuthorship: Sigwart; nomenclaturalCode: ICZN; **Location:** higherGeography: Izu-Ogasawara Trench; country: Japan; verbatimLocality: Izu-Ogasawara Trench, West of KEO; verbatimDepth: 5506; minimumDepthInMeters: 5506; maximumDepthInMeters: 5506; verbatimCoordinates: 32°34.6760' N, 143°46.1235' E; verbatimLatitude: 32°34.6760' N; verbatimLongitude: 143°46.1235' E; decimalLatitude: 32.577933; decimalLongitude: 143.768725; **Identification:** identifiedBy: Juila Sigwart; dateIdentified: 2024; **Event:** samplingProtocol: suction sampler; eventDate: 2024-05; year: 2024; month: 5; habitat: woodfall; **Record Level:** language: en; institutionID: 16946ec1-8db3-45b8-b084-7644384cc5f5; basisOfRecord: PreservedSpecimen
- **Type status:** Paratype. **Occurrence:** catalogNumber: SMF 383144; recordedBy: Chong Chen; individualCount: 1; lifeStage: adult; occurrenceID: BE0766CA-8BAE-5F9C-A62D-2E724051E8B1; **Taxon:** scientificName: Ferrieraella populi; kingdom: Animalia; phylum: Mollusca; class: Polyplacophora; order: Lepidopleurida; family:  Ferreiraellidae; genus:  Ferreiraella; specificEpithet: populi; taxonRank: species; scientificNameAuthorship: Sigwart; nomenclaturalCode: ICZN; **Location:** higherGeography: Izu-Ogasawara Trench; country: Japan; verbatimLocality: Izu-Ogasawara Trench, West of KEO; verbatimDepth: 5506; minimumDepthInMeters: 5506; maximumDepthInMeters: 5506; verbatimCoordinates: 32°34.6760' N, 143°46.1235' E; verbatimLatitude: 32°34.6760' N; verbatimLongitude: 143°46.1235' E; decimalLatitude: 32.577933; decimalLongitude: 143.768725; **Identification:** identifiedBy: Juila Sigwart; dateIdentified: 2024; **Event:** samplingProtocol: suction sampler; eventDate: 2024-05; year: 2024; month: 5; habitat: woodfall; **Record Level:** language: en; institutionID: 16946ec1-8db3-45b8-b084-7644384cc5f5; basisOfRecord: PreservedSpecimen
- **Type status:** Paratype. **Occurrence:** catalogNumber: NSMT-Mo 79868; recordedBy: Chong Chen; individualCount: 1; lifeStage: adult; occurrenceID: 34B3141A-0824-520A-ACD9-7E9BD4236BB2; **Taxon:** scientificName: Ferrieraella populi; kingdom: Animalia; phylum: Mollusca; class: Polyplacophora; order: Lepidopleurida; family:  Ferreiraellidae; genus:  Ferreiraella; specificEpithet: populi; taxonRank: species; scientificNameAuthorship: Sigwart; nomenclaturalCode: ICZN; **Location:** higherGeography: Izu-Ogasawara Trench; country: Japan; verbatimLocality: Izu-Ogasawara Trench, West of KEO; verbatimDepth: 5506; minimumDepthInMeters: 5506; maximumDepthInMeters: 5506; verbatimCoordinates: 32°34.6760' N, 143°46.1235' E; verbatimLatitude: 32°34.6760' N; verbatimLongitude: 143°46.1235' E; decimalLatitude: 32.577933; decimalLongitude: 143.768725; **Identification:** identifiedBy: Juila Sigwart; dateIdentified: 2024; **Event:** samplingProtocol: suction sampler; eventDate: 2024-05; year: 2024; month: 5; habitat: woodfall; **Record Level:** language: en; institutionID: d08b12cb-dc48-4de3-b08a-47ea57591abe; basisOfRecord: PreservedSpecimen
- **Type status:** Paratype. **Occurrence:** catalogNumber: NSMT-Mo 79869; recordedBy: Chong Chen; individualCount: 1; lifeStage: adult; occurrenceID: 738029B9-DFC8-5ACE-A76B-8831E2718B1F; **Taxon:** scientificName: Ferrieraella populi; kingdom: Animalia; phylum: Mollusca; class: Polyplacophora; order: Lepidopleurida; family:  Ferreiraellidae; genus:  Ferreiraella; specificEpithet: populi; taxonRank: species; scientificNameAuthorship: Sigwart; nomenclaturalCode: ICZN; **Location:** higherGeography: Izu-Ogasawara Trench; country: Japan; verbatimLocality: Izu-Ogasawara Trench, West of KEO; verbatimDepth: 5506; minimumDepthInMeters: 5506; maximumDepthInMeters: 5506; verbatimCoordinates: 32°34.6760' N, 143°46.1235' E; verbatimLatitude: 32°34.6760' N; verbatimLongitude: 143°46.1235' E; decimalLatitude: 32.577933; decimalLongitude: 143.768725; **Identification:** identifiedBy: Juila Sigwart; dateIdentified: 2024; **Event:** samplingProtocol: suction sampler; eventDate: 2024-05; year: 2024; month: 5; habitat: woodfall; **Record Level:** language: en; institutionID: d08b12cb-dc48-4de3-b08a-47ea57591abe; basisOfRecord: PreservedSpecimen

#### Description

Holotype 17 × 9.5 mm, oval. Overall colour of valves cream, off-white, marked with black spots of mineral deposits on valve diagonal.

**Figure 1.**
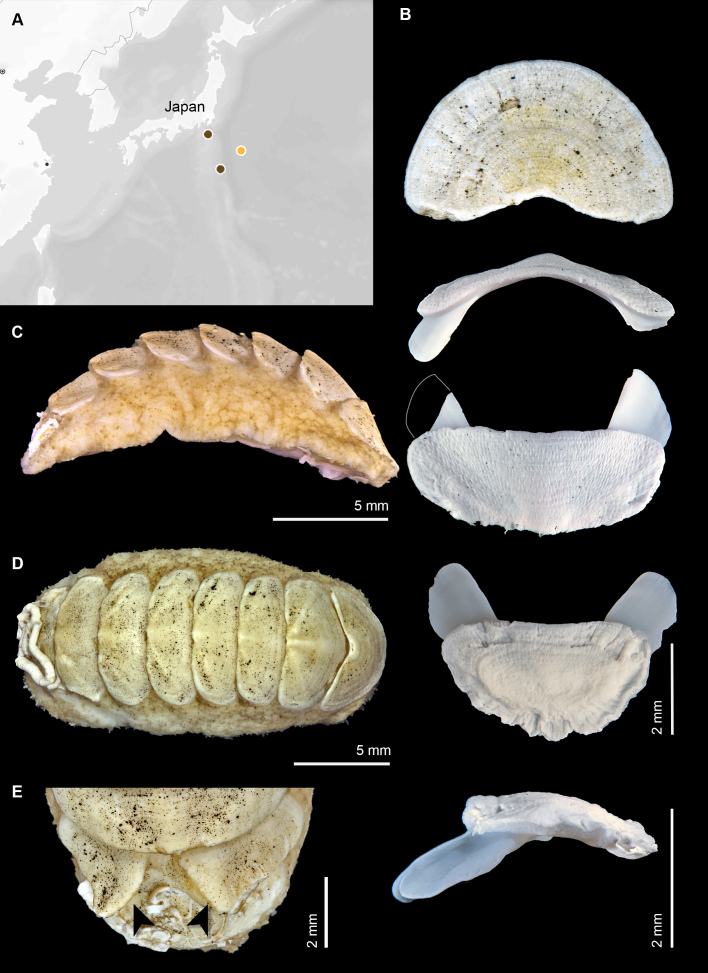
*Ferreiraella populi* sp. nov. **A** Schematic, non-scaled outline map provided for geographic orientation to the type localities of Japanese species in the genus, including the new species (coloured dot). **B** Valves from holotype (SMF 383139) showing (from top to bottom) valve I, valve III (anterior view), valve III (dorsal views), valve VIII and valve VIII in lateral view; valve VIII was cleaned to remove epibionts prior to photographing. **C** Holotype SMF 383139, lateral view prior to dissection. **D** Paratype SMF 383138, dorsal view. **E** Paratype SMF 383142, dorsal view of posterior part showing split-valve teratology on valve VII.

**Figure 2.**
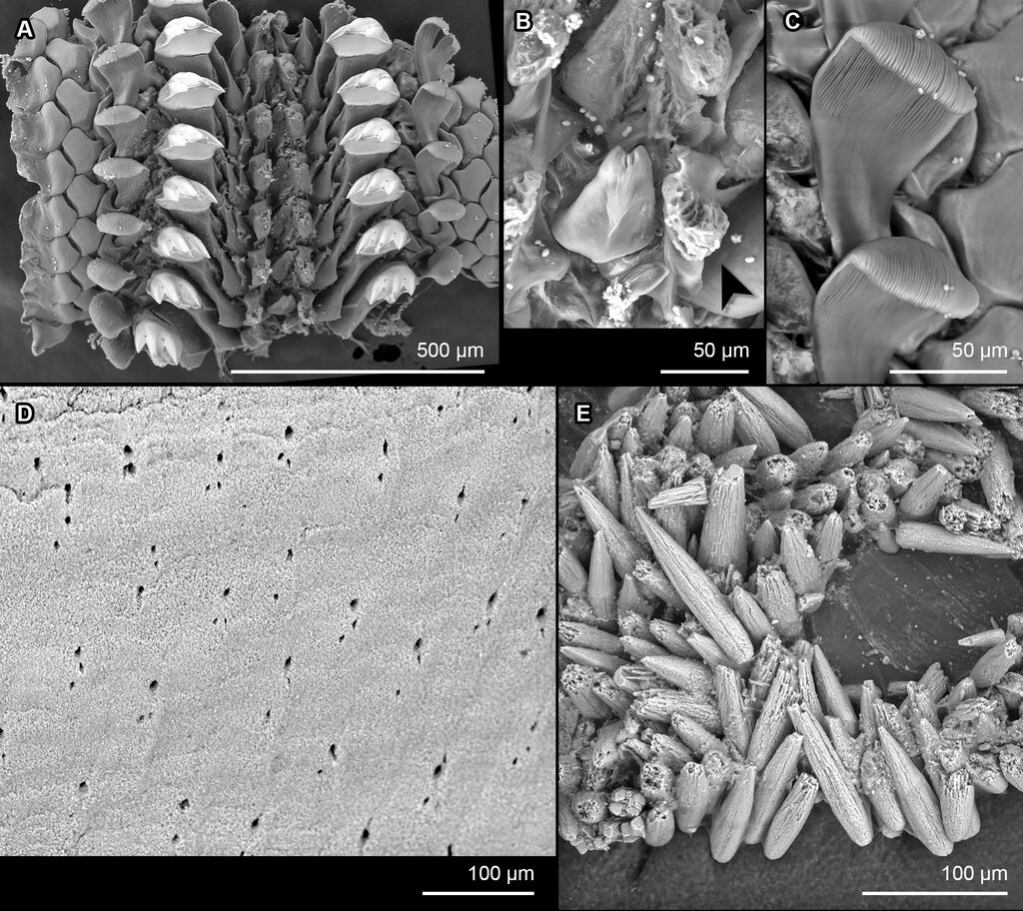
*Ferreiraella populi* sp. nov. holotype (SMF 383139), SEM micrographs: **A** Radula. **B** Radula first (inner) lateral tooth indicated with arrowhead. **C** Radula major uncinus (sweeper) tooth. **D** Aesthete pores, shown from the anterior central margin of valve III. **E** Girdle scales (perinotum).

Valves rounded, not elevated (dorsal elevation ratio nearly flat in valve III, Fig. 1A). Head valve semicircular. Intermediate valves rectangular, lateral margins rounded but relatively straight, anterior margin slightly convex, posterior margin paralleling anterior margin, apex not projecting. Lateral areas slightly raised, aligned with posterior edge of underlying apophysis, visible from contrasting mineral deposits that are mainly seen on either side of the diagonal. Tail valve smaller than head valve, flattened, mucro central, posterior slope flat but margin slightly crenulate (Fig. 1A and B). Tegmentum smooth, covered with thin tissue and minor apical projections from aesthete pores. Aesthetes irregularly arranged in bundles of 1 megalaesthete and approximately 2 micraesthetes (Fig. 2A and D). Aesthete bundles typically widely separated with macraesthete anterior and two micraesthetes trailing to posterior.

Articulamentum white, well developed, without insertion plate or callus, apophyses moderately narrow, triangular in the intermediate valves and tail valve. Jugal sinus wide.

Central tooth of radula small, bud-like, first (inner) lateral teeth with compact brush-like projections (Fig. 2A and B). Second (major) lateral teeth with flattened, tridentate mineralised cusps. Major uncinal (sweeper) teeth broad, spoon-shaped, but comb-like blade, similar in width and height to major lateral teeth (Fig. 2C). Radula of the holotype measured 6.1 mm with 53 rows of fully mineralised teeth.

Girdle very wide, dorsally densely covered with oblong spicule-scale, only one type observed, but longer towards the valve interstices (80-120 × 20-25 μm). Scales distally pointed, without sculpture, smooth, but rough in texture, oval in cross section (Fig. 2E). Ventral side of girdle naked.

Gills 18-20 on each side (18 in holotype SMF 383139; 20 in paratype SMF 383138), smaller to the anterior, extending from valve V to the anus. Schwabe organ not visible.

#### Diagnosis

Animal medium, type specimens up to approximately 25 mm long. Overall colour off-white. Shell rounded, valves not beaked, mucro of tail valve central and not raised; tail valve smaller than head valve. Tegmentum smooth; aesthetes in irregular bundles of 1 megalaesthete and 2 micraesthetes, with protruding caps. Girdle densely covered with oblong spicule-scales. Major lateral teeth of radula with relatively flattened, tridentate cusps. Major uncinus comb-like, not higher than the major lateral. Eighteen to twenty gills on each side.

#### Etymology

Species epithet "*populi*", Latin singular noun in the genetive case, meaning "of the people". This name was suggested 11 different times from over 8,000 total contributions to the public naming competition.

#### Distribution

Only known from the type locality, woodfall on the abyssal plain in the Northwestern Pacific, about 150 km east of the Ogasawara Trench axis, off Japan, 32°34.6760 N, 143°46.1235 E, 5506 m depth.

#### Ecology

Endemic to sunken wood.

#### Type material

Type specimens are deposited at the Senckenberg Research Institute and Natural History Museum Frankfurt, Germany and the National Museum of Nature and Science, Japan. Holotype (SMF 383139) partially disarticulated, consisting of mounts of shell, perinotum and radula, originally fixed in formalin; paratype 1 used for DNA barcoding (SMF 383142), three additional paratypes originally preserved in 99% ethanol (SMF 383143, SMF 383144, NSMT-Mo 79868), four paratypes originally fixed in formalin (NSMT-Mo 79869, SMF 383138, SMF 383140, SMF 383141).

#### Notes

Molecular data are available only for two other species in this genus, and the COI barcode region from the new species (GenBank accession number PX804841) is only 88% identical with *Ferreiraella plana* (GenBank accession number HQ907844) and 87% identical with *F. xylophaga karenae* (GenBank accession numbers HQ907845, HQ907846) based on BLAST comparison (https://blast.ncbi.nlm.nih.gov/).

The new species can be distinguished from all other members of the genus by its unusually small tail valve, and having aesthetes with fewer pores in a bundle and with bundles that are unusually stretched with longer separation between a macraesthete and corresponding micraesthetes. The new species has the distinctive comb-form major uncinus “sweeper” teeth that are diagnostic for *Ferreiraella*, but they are less enlarged in the new species than other members of the genus. The compact, brush-tipped inner lateral teeth are also described from other members of the genus and here are apparently soft and complex, as the dehydrated teeth form a cap like a morel.

The new species is most similar in superficial appearance to *F. tsuchidai* Saito, 2006, which occurs at similar depth (type locality 5567 m) in the Philippine Basin, in terms of the valve shape and elevation. Specimens of *F. tsuchidai* and *F. charazata* Sigwart, 2025 (Senckenberg Ocean Species Alliance (SOSA) et al. 2025), like the new species, have been observed to frequently host epibiotic serpulid tubeworms on their tail valves (Saito 2006, Senckenberg Ocean Species Alliance (SOSA) et al. 2025). The new species represents the third species of *Ferreiraella* found in Japan, after *F. takii* (Wu & Okutani, 1984) and *F. soyomaruae* (Wu & Okutani, 1984) were described from shallower depths of 1250 m (*F. takii*) and 3100 m (*F. soyomaruae*), and the new species is more closely similar to *F. takii*. *Ferreiraella takii* differs from the new species in its valve shape, with more convex anterior margin of the intermediate valves compared to straighter in the new species, and the size of the tail valves, which is distinctly smaller than the head valve in the new species, whereas *F. takii* has head and tail valve of about the same size. Interestingly, the radula is also different, with a shorter radula (5.5 mm in *F. takii*) with apparently denser arrangement of teeth in *F. takii* (over 54 rows of teeth in the new species, but 68 rows in a shorter radula in *F. takii* (Wu and Okutani 1984). Finally, *F. takii* has several types of girdle spicules, where only one was observed in the new species.

Paratype 1 (SMF 383142) of the new species shows a valve teratology on valve VII (Fig. 1E) with the valve split on the mid-line and growing in two slightly separated segments. Valve splitting has been observed in other species, although not frequently (Dell'Angelo and Schwabe 2010) and has been described with tomographic reconstruction in the shallow water species *Acanthopleura granulata* (Gmelin, 1791) (Kingston et al. 2019).

Deep sea deposits of sunken wood provide habitat for diverse chitons species including two endemic families, Nierstraszellidae and Ferreiraellidae. Evidence from gut-associated bacteria in other wood-endemic chitons indicates that they are secondary consumers, not directly feeding on the wood (Duperron et al. 2012), but this has never been tested for *Ferreiraella*.

### Notoplax madagascariensis

Vončina & Schwabe sp. nov.

C7371443-D5BE-5D30-9826-BF8FFACE82C5

82FFB7F4-376C-4170-8C68-5B33DD08AA75

#### Materials

- **Type status:** Holotype. **Occurrence:** catalogNumber: MNHN-IM-2007-38001; recordedBy: leg. P. Bouchet and Y. Kantor; individualCount: 1; lifeStage: adult; occurrenceID: 7F175B10-3934-56A0-BB9E-F88BBACE7A6A; **Taxon:** scientificName: Notoplax madagascariensis Vončina & Schwabe; kingdom: Animalia; phylum: Mollusca; class: Polyplacophora; order: Chitonida; family: Acanthochitonidae; genus: Notoplax; specificEpithet: madagascariensis; taxonRank: species; scientificNameAuthorship: Vončina & Schwabe; nomenclaturalCode: ICZN; **Location:** higherGeography: Indian Ocean; country: Madagascar; locality: Madagascar, off Mahajamba Bay; minimumDepthInMeters: 90; maximumDepthInMeters: 257; verbatimLatitude: 14°53'S; verbatimLongitude: 46°56'E; **Identification:** identifiedBy: Katarzyna Vončina; dateIdentified: 2024; **Event:** eventDate: 2009-07-07; habitat: on sponges; eventRemarks: Expedition: MIRIKY, Station: DW3245, Ship: MIRIKY; **Record Level:** language: en; basisOfRecord: PreservedSpecimen
- **Type status:** Paratype. **Occurrence:** catalogNumber: MNHN-IM-2009-16615; recordedBy: leg. P. Bouchet and Y. Kantor; individualCount: 1; lifeStage: adult; occurrenceID: 2398476C-3047-5C01-B48F-537F0DF27469; **Taxon:** scientificName: Notoplax madagascariensis Vončina & Schwabe; kingdom: Animalia; phylum: Mollusca; class: Polyplacophora; order: Chitonida; family: Acanthochitonidae; genus: Notoplax; specificEpithet: madagascariensis; taxonRank: species; scientificNameAuthorship: Vončina & Schwabe; nomenclaturalCode: ICZN; **Location:** higherGeography: Indian Ocean; country: Madagascar; locality: Madagascar, in front of Nazendry Bay; minimumDepthInMeters: 48; maximumDepthInMeters: 139; verbatimLatitude: 14°29'S; verbatimLongitude: 47°27'E; **Identification:** identifiedBy: Katarzyna Vončina; dateIdentified: 2024; **Event:** eventDate: 2009-07-06; habitat: on sponges; eventRemarks: Expedition: MIRIKY, Station: DW3238, Ship: MIRIKY; **Record Level:** language: en; basisOfRecord: PreservedSpecimen
- **Type status:** Paratype. **Occurrence:** catalogNumber: SMF 383079; recordedBy: leg. Bouchet, Rosado and Strong; individualCount: 1; lifeStage: adult; otherCatalogNumbers: MNHN-IM-2007-38323; occurrenceID: 319ABA30-B2B2-53E8-B9DF-E067D162E3A9; **Taxon:** scientificName: Notoplax madagascariensis Vončina & Schwabe; kingdom: Animalia; phylum: Mollusca; class: Polyplacophora; order: Chitonida; family: Acanthochitonidae; genus: Notoplax; specificEpithet: madagascariensis; taxonRank: species; scientificNameAuthorship: Vončina & Schwabe; nomenclaturalCode: ICZN; **Location:** higherGeography: Indian Ocean; locality: Mozambique Channel, Almirante Leite Bank; minimumDepthInMeters: 87; maximumDepthInMeters: 90; verbatimLatitude: 26°12'S; verbatimLongitude: 35°03'E; **Identification:** identifiedBy: Katarzyna Vončina; dateIdentified: 2024; **Event:** eventDate: 2009-04-16; eventRemarks: Expedition: MAINBAZA, Station: DW3168, Ship: Vizconde de Eza; **Record Level:** language: en; basisOfRecord: PreservedSpecimen

#### Description

**Figure 3.**
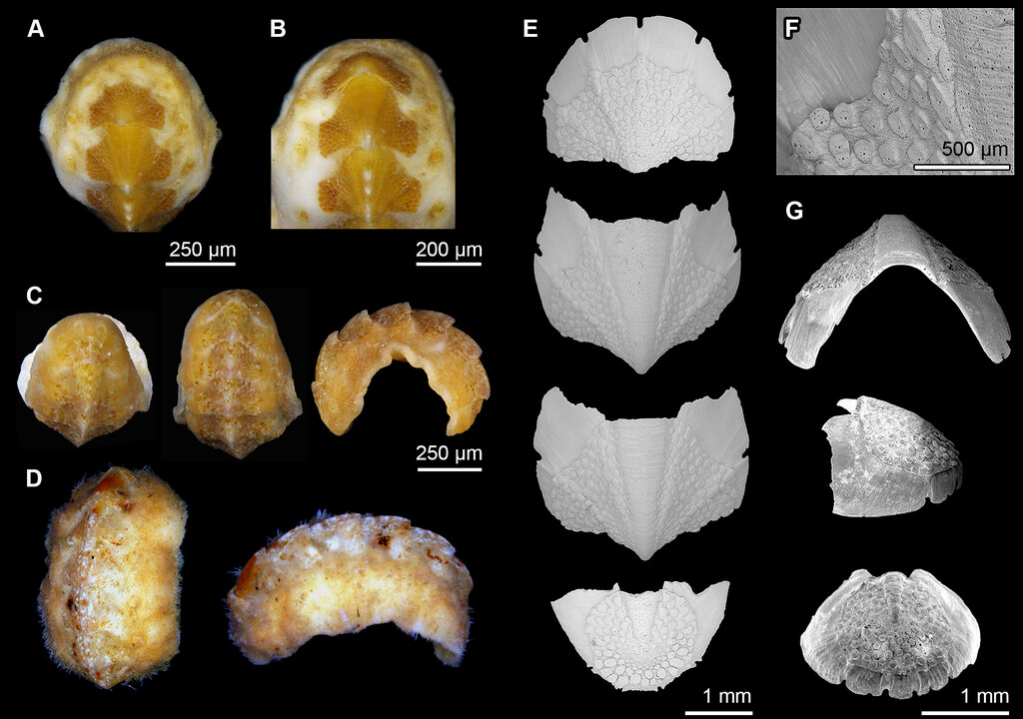
*Notoplax madagascariensis* sp. nov. **A, B** Holotype (MNHN-IM-2007-38001), dorsal view. **C** Paratype (SMF 383079), dorsal and lateral view. **D** Paratype (MNHN-IM-2009-16615), dorsal and lateral view, respectively. **E** Holotype (MNHN-IM-2007-38001) valves from top to bottom: Valves I, II, III, VIII, dorsal view. **F** Holotype (MNHN-IM-2007-38001) aesthetes on valve II in the anterior part of lateropleural area. **G** Holotype (MNHN-IM-2007-38001) valves: intermediate valve in rostral view, valve VIII in lateral and caudal view.

Body of small size (approximate size due to curled up specimens; holotype MNHN-IM-2007-38001: 14.5 x 6 mm; paratype MNHN-IM-2009-16615: 9.1 mm; paratype SMF 383079: 15.5 x 5 mm), oval, carinated, highly elevated (valve elevation = 0.56), side slopes slightly concave; valves solid, beaked (Fig. 3). Tegmentum granulated, orange, ochraceous; girdle broad, encroaching at valve sutures, orange or white with orange blotches (Fig. 3).

Head valve semicircular with five radiating rows of slightly larger, raised granules; posterior margin almost straight with a small apex. Intermediate valves roughly pentagonal in shape, wider than long, highly elevated, anterior margin straight, side margins concave, posterior margin concave and beaked; jugum narrow and wedge-shaped, smooth; lateral areas separated from pleural areas by radiating rows of larger granules on slightly elevated rib (Fig. 3). Tail valve round in outline; mucro not raised, located slightly behind the centre; posterior slope almost straight to slightly concave (Fig. 3). Granules on tegmentum uniformly, rather densely distributed, oval, raised, flat-topped to slightly concave, slightly larger on radial ribs; each granule with 1-2 megalaesthete and 5-12 micraesthetes around them; micraesthetes present in tegmental plain (Fig. 3F). Articulamentum well-developed, white, translucent-white at the edges and orange-brown in the central part, apophyses wide, long, sharply-edged to slightly rounded in intermediate valves, truncate in tail valve, insertion plates long in head valve, moderately long in intermediate valves, slit formula 5/1/4-5, slits U-shaped, and accompanied by a shallow groove extending from the edge to tegmentum, no slit rays (Fig. 3E and G).

**Figure 4.**
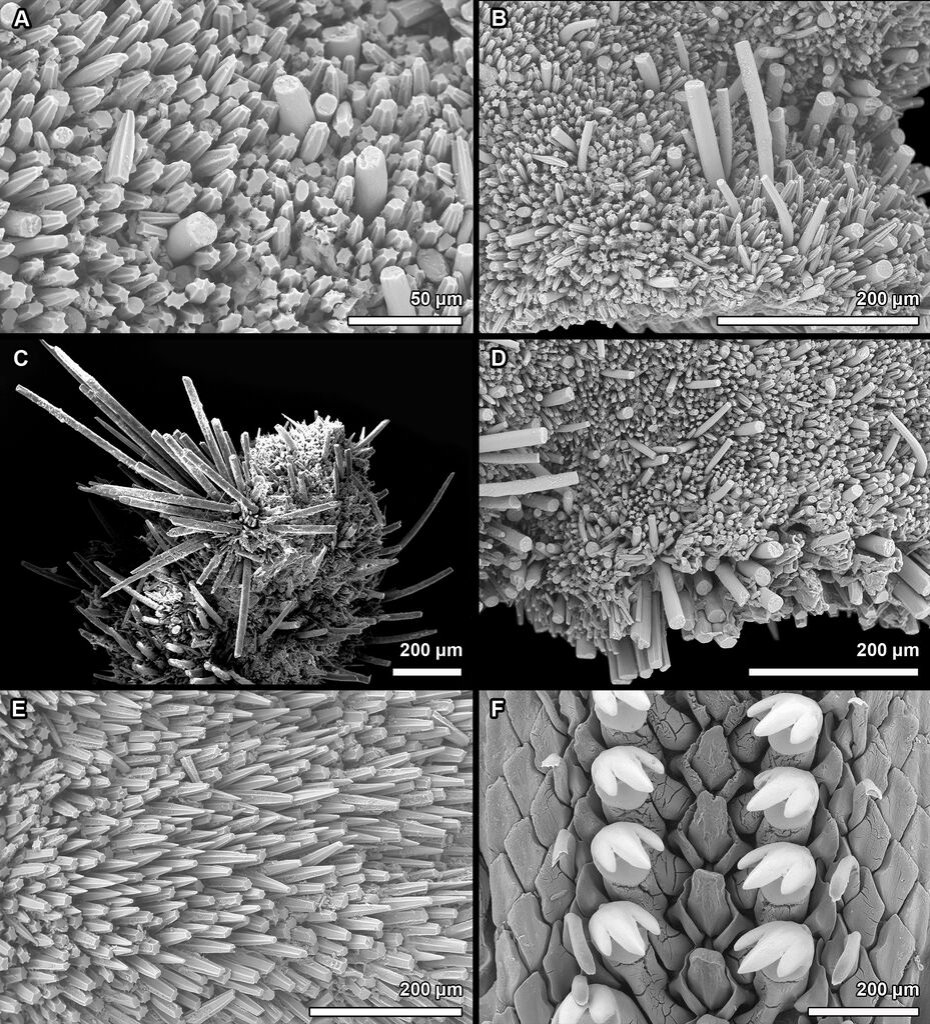
*Notoplax madagascariensis* sp. nov., holotype (MNHN-IM-2007-38001). **A, B** Dorsal girdle spicules. **C** Sutural tuft and dorsal spicules. **D** Marginal and dorsal spicules. **E** Ventral spicules. **F** Central portion of radula.

Girdle fleshy, wide, white with orange blotches or uniformly orange, dorsally covered with three kinds of spicules: short, sharply pointed, strongly ribbed spicules, L: 42–60 μm (mean = 53 μm, n = 11), W: 7–10 μm (mean = 8.9 μm, n = 11); intermingled with longer, smooth spicules that are round in cross-section, finely striated at the posterior end and slightly bent, L: 120–150 μm (mean = 135 μm, n = 2), W: 13–14 μm (mean = 13.5 μm, n = 2); and sparsely distributed, long, thick, smooth spicules, L: 250–400 μm (mean = 325 μm, n = 2), W: 20 μm (n = 2) (Fig. 4A-D). Sutural tufts up to 30 straight, smooth needles up to 700 μm x 30 μm, mixed with much shorter, sharp needles (Fig. 4C); marginal spicules similar to those of sutural tufts (Fig. 4D). Ventral spicules similar to strongly ribbed dorsal spicules but longer L: 90–195 μm (mean = 124 μm, n = 13), W: 14–22 μm (mean =18, n = 13) (Fig. 4E).

Radula of holotype ca. 6.4 mm long, with 32 rows of teeth, of which 27 are mineralised; radula of paratype MNHN-IM-2009-16615 54.2 mm long, with 34 rows of teeth, 26 mineralised; arrangement of teeth asymmetrical, central tooth rectangular, asymmetrical with bilobed base; first lateral tooth thin, weakly notched antero-dorsal corner; major lateral tooth with tricuspid head, denticles pointed, central denticle longer than others; major uncinal tooth long, narrow (Fig. 4F).

Gills merobranchial, 6 ctenidia per side in the MNHN paratype, in other specimens completely covered by mantle fold.

#### Diagnosis

Animal small (15 mm or less), body oval, valves carinated and highly elevated, beaked, tegmentum covered with oval granules, colour orange to ochraceous. Head valve semicircular with five radiating rows of slightly enlarged granules; intermediate valves pentagonal, with narrow, smooth jugum; tail valve rounded, mucro subcentral. Articulamentum well developed, slit formula 5/1/4–5. Girdle broad, fleshy, dorsally covered with short and strongly ribbed spicules, intermingled with longer spicules, 18 tufts of spicules at the sutures. Radula asymmetrical.

#### Etymology

The specific epithet *madagascariensis* is derived from Madagascar, referring to the locality where the species was first discovered. The Latin suffix -ensis denotes origin or association, thus the name signifies “from Madagascar”.

#### Distribution

At present known only from the type locality, north-western Madagascar, off Mahajamba Bay, Indian Ocean at 14°53′ S, 46°56′ E, collected at 87–257 m depth.

#### Ecology

Found in association with sponges.

#### Type material

Holotype (MNHN-IM-2007-38001) now partly disarticulated: mounts of shells (valves I, II, III, VIII) and parts of girdle and radula mounted on three SEM stubs, respectively, and the vial with the specimen stored in 96% ethanol; one paratype (MNHN-IM-2009-16615) now partly disarticulated: mounts of shells (valves I, II, VIII) and parts of girdle and radula mounted on two SEM stubs, respectively, and the vial with the specimen stored in 96% ethanol; one paratype (SMF 383079) stored in 96% ethanol.

#### Differential Diagnosis

*Notoplax madagascariensis* sp. nov. resembles several species of *Notoplax* and *Leptoplax* in valve outline, sculpture, or girdle ornamentation (especially *L. tongkingi*), but differs from:

*Notoplax aenigma* Iredale & Hull, 1923 by mucro located less posteriorly with straight pms slope (vs. posterior mucro with concave pms slope in *N. aenigma*), and more sparsely distributed, smaller granules (Iredale and Hull 1923).

*Notoplax arabica* Kaas and van Belle, 1988 by denser, smaller and more oval granules, and much longer intermediate valves (Kaas and Belle 1988).

*Notoplax formosa* Reeve, 1847 by more sparsely arranged tegmental granules, missing sulci (vs. jugum separated by a series of sulci in *N. formosa*), straight pms slope (vs. concave pms slope in *N. formosa*) (Reeve 1847).

*Notoplax rostellata* Kaas, 1990 by oval granules with 1–2 megalaesthetes surrounded by 5-12 micraesthetes (vs. round granules with one megalaesthete surrounded by 1–3 micraesthetes), more slender and shorter dorsal short spicules (up to 60 x 10 μm in *N. madagascariensis* sp. nov. vs. 160 x 12 in *N. rostellata*), mucro located less posteriorly (Kaas 1990).

*Notoplax tateyamaensis* Wu & Okutani, 1995 by smaller size (up to 15.5 mm *N. madagascariensis* sp. nov. vs. 28 mm in *N. tateyamaensis*), strongly ribbed dorsal and ventral spicules (vs. smooth in *N. tateyamaensis*), mucro located less posteriorly with straight pms slope (vs. posterior mucro with concave pms slope in *N. tateyamaensis*) (Wu and Okutani 1995).

*Leptoplax richardi* (Kaas, 1990) by larger, more oval tegmental granules, narrower jugum, missing sulci (vs. jugum separated by a series of sulci in *L. richardi*), mucro located less posteriorly, and strongly ribbed dorsal and ventral spicules (vs. smooth in *L. richardi*) (Kaas 1990).

*Leptoplax tongkingi* (Sirenko & Saito, 2017) by having more elevated intermediate valves, pentagonal outline of intermediate valves (vs. rectangular in *L. tongkingi*), more densely arranged and smaller granules, narrower jugum, and clear separation of size between megalaesthetes and micraesthetes (vs. almost indistinguishable in *L. tongkingi*) (*Sirenko and Saito 2017*).

*Leptoplax unica* (Nierstrasz, 1905) by having more elevated intermediate valves, less reduced tegmentum, tegmental granules with 1–2 megalaesthetes surrounded by micraesthetes (vs. only megalaesthete in *L. unica*), asymmetrical radula (vs. symmetrical in *L. unica*), strongly ribbed dorsal and ventral spicules (vs. smooth in *L. unica*) (Nierstrasz 1905).

Additionally, previously illustrated specimens by Leloup (1981b) from Tuléar (Madagascar), originally assigned to *Acanthochitona* and later transferred to *Notoplax* by Kaas (1986), show valve outlines and sculpture similar to *N. madagascariensis*; however, the girdle ornamentation differs markedly (Leloup 1981b: fig. 3).

#### Notes

The placement of *Notoplax madagascariensis* sp. nov. is tentative due to its combination of morphological characters that overlap with both *Notoplax* and *Leptoplax* (sensu Sirenko and Saito (2017)). The separation between *Notoplax* and *Leptoplax* was historically confounded until Gowlett-Holmes (1991) provided a diagnosis for *Notoplax*, proposing also that *Notoplax* sensu stricto is endemic to Australia and New Zealand. Later, Sirenko and Saito (2017) amended the diagnoses for both genera, with further revision of *Leptoplax* by Sirenko (2024), and expanded its range in the Indo-Pacific.

Morphologically, the new species exhibits larger tegmental granules along the valve diagonal and radula features typical of *Notoplax* (sensu Sirenko and Saito (2017)). The girdle does not completely encroach between the valves, the articulamentum is moderately developed, and strongly ribbed dorsal and ventral spicules are present, traits reminiscent of *Leptoplax* (Sirenko 2024). Moreover, the diagonal granules are not prominent and are sometimes difficult to observe in the examined specimens. Taken together, these observations support the hypothesis that *N. madagascariensis* sp. nov. represents a lineage distinct from currently recognised genera within Cryptoplacoidea, pending a formal systematic revision.

### Placiphorella granulosa

Vončina sp. nov.

E371C4FE-A78A-5523-B581-E163DCCFDA04

D40727CD-4F44-4F01-99FC-217E3ECC2C88

#### Materials

- **Type status:** Holotype. **Occurrence:** catalogNumber: MNHN-IM-2019-34864; recordedBy: leg. Samadi and Corbari; individualCount: 1; lifeStage: adult; occurrenceID: 6AC81AF3-7B6E-5CB1-87C0-7FDB7C2D4E30; **Taxon:** scientificName: Placiphorella granulosa Vončina; kingdom: Animalia; phylum: Mollusca; class: Polyplacophora; order: Chitonida; family: Mopaliidae; genus: Placiphorella; specificEpithet: granulosa; taxonRank: species; scientificNameAuthorship: Vončina; nomenclaturalCode: ICZN; **Location:** higherGeography: Pacific Ocean; country: Papua New Guinea; locality: Papua New Guinea, Vitiaz Strait; minimumDepthInMeters: 860; maximumDepthInMeters: 880; verbatimLatitude: 05°59'S; verbatimLongitude: 147°39'E; **Identification:** identifiedBy: Katarzyna Vončina; dateIdentified: 2024; **Event:** eventDate: 2010-10-07; eventRemarks: Expedition: BIOPAPUA, Station: CP3724, Ship: Alis; **Record Level:** language: en; basisOfRecord: PreservedSpecimen; **Material Entity:** associatedSequences: https://www.ncbi.nlm.nih.gov/nuccore/PX804842
- **Type status:** Paratype. **Occurrence:** catalogNumber: SMF 383080; recordedBy: leg. Samadi and Corbari; individualCount: 1; lifeStage: adult; otherCatalogNumbers: MNHN-IM-2019-35216; occurrenceID: 2806D9FE-C85C-5A12-B42A-3CACAD7E87A5; **Taxon:** scientificName: Placiphorella granulosa Vončina; kingdom: Animalia; phylum: Mollusca; class: Polyplacophora; order: Chitonida; family: Mopaliidae; genus: Placiphorella; specificEpithet: granulosa; taxonRank: species; scientificNameAuthorship: Vončina; nomenclaturalCode: ICZN; **Location:** higherGeography: Pacific Ocean; country: Papua New Guinea; locality: Papua New Guinea, Vitiaz Strait; minimumDepthInMeters: 860; maximumDepthInMeters: 880; verbatimLatitude: 05°59'S; verbatimLongitude: 147°39'E; **Identification:** identifiedBy: Katarzyna Vončina; dateIdentified: 2024; **Event:** eventDate: 2010-10-07; eventRemarks: Expedition: BIOPAPUA, Station: CP3724, Ship: Alis; **Record Level:** language: en; basisOfRecord: PreservedSpecimen

#### Description

**Figure 5.**
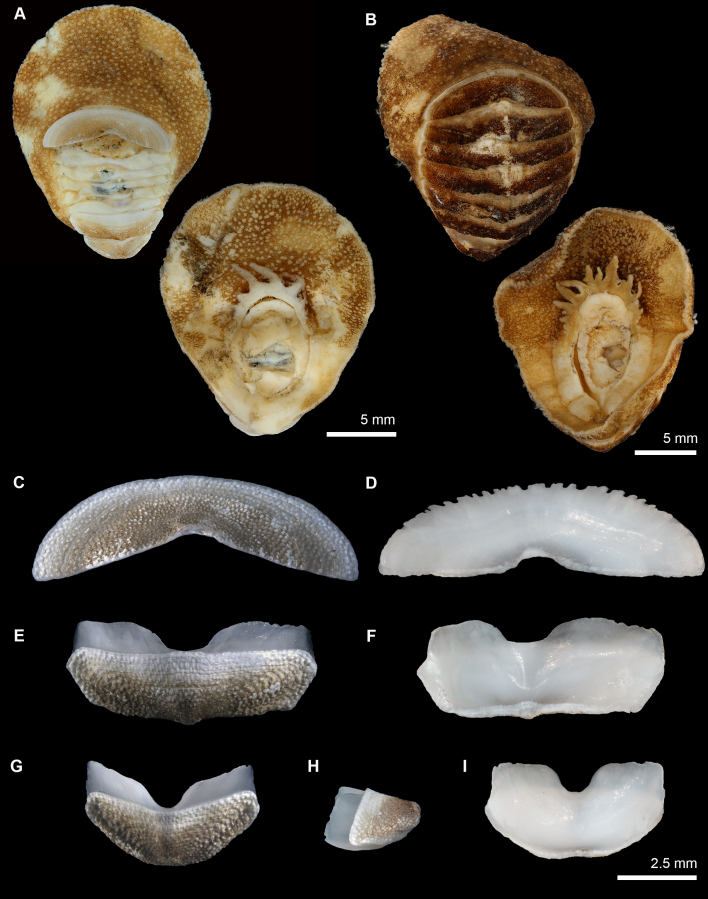
*Placiphorella granulosa* sp. nov. **A** Holotype (MNHN-IM-2019-34864), dorsal and ventral view, respectively. **B** Paratype (SMF 383080), dorsal and ventral view, respectively. **C-G, I** holotype (MNHN-IM-2019-34864) valves: dorsal (left) and ventral (right) view of valves I, VII, VIII. **H** lateral view of valve VIII.

Body of medium size (holotype MNHN-IM-2019-34864: 18 x 15 mm, paratype SMF 383080: ca. 19.5 x 16 mm – specimen curled up; both specimens measured from the ventral side), broadly oval, subcarinated, moderately elevated (valve elevation = 0.35), side slopes straight; valves solid, not beaked or with small, not pronounced apex (Fig. 5). Tegmentum densely granulated, white-yellow or brown; girdle broadly expanded anteriorly, light-brown with white patches (Fig. 5).

**Figure 6.**
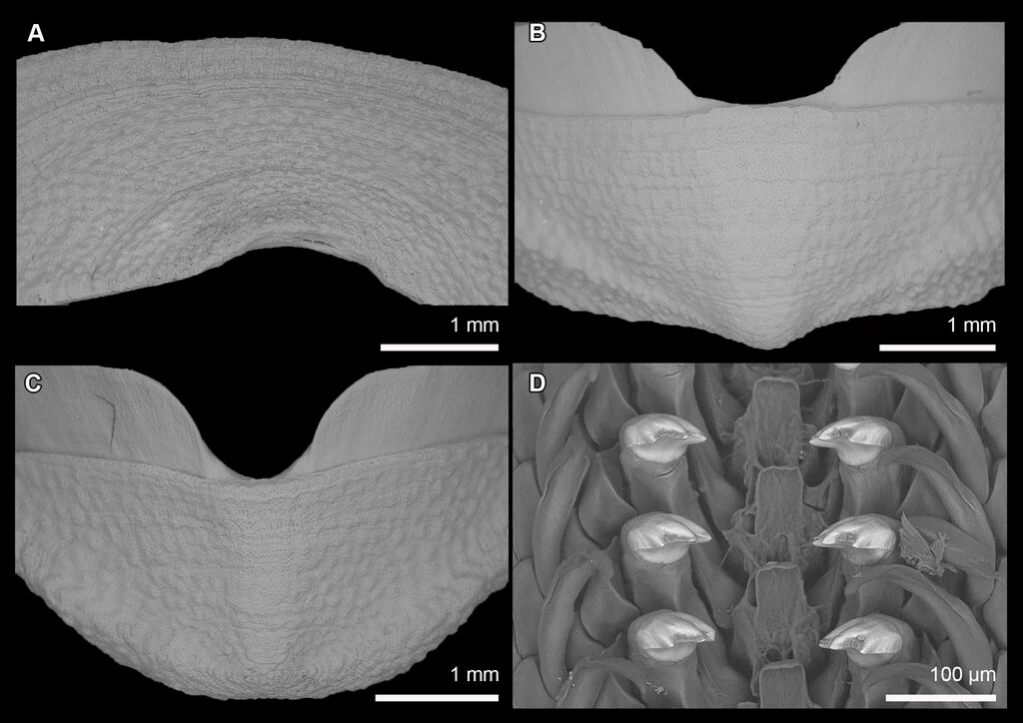
*Placiphorella granulosa* sp. nov., holotype (MNHN-IM-2019-34864). **A–C** Details of dorsal surface of valve I, VII, VIII, respectively. **D** Central portion of radula.

Head valve crescent-shaped, front slope concave, posterior margin very widely V-shaped, with a small median notch, tegmentum granulated, some inconspicuous concentric growth lines (Figs 5, 6A). Intermediate valves broadly rectangular, much wider than long, front margin almost straight, weakly projected forward at jugal part; side margins rounded, hind margin straight to weakly convex, apex weakly indicated, tegmentum granulated, lateral areas raised, bordered by weakly indicated diagonal ribs (Figs 5, 6B). Tail valve small, roughly trapezoidal in outline, with front margin concave and rounded posterior margin, mucro raised, terminal, overhanging, antemucronal area straight, postmucronal area convex, tegmentum granulated with two ribs separating the ante- and postmucronal areas (Figs 5, 6C).

Articulamentum strongly developed, white, valves calloused, apophyses very wide, short, slightly rounded to subtrapezoidal, paralleled in the tail valve, separated by a jugal sinus, insertion plates short, slit formula 19/1/sinus, slits shallow, no slit rays, teeth thick, bilobed and crenulated (Fig. 5C-I).

**Figure 7.**
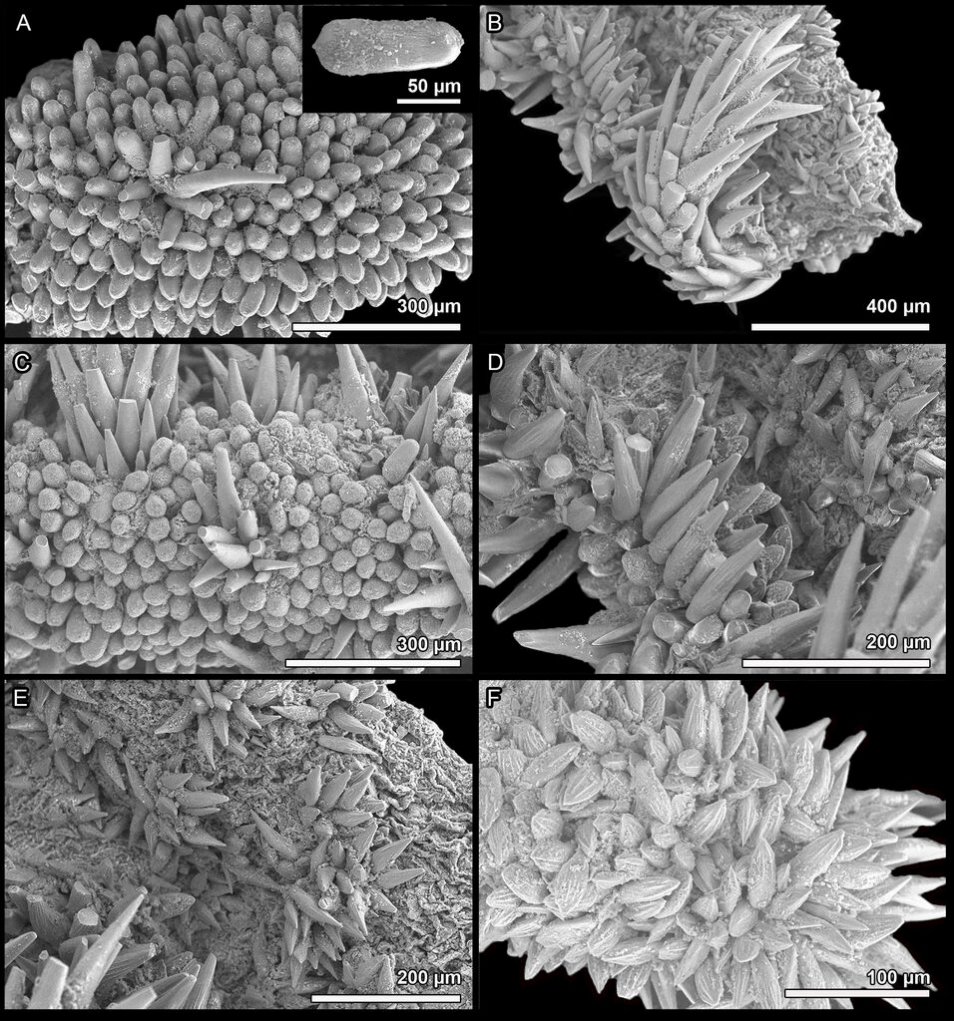
*Placiphorella granulosa* sp. nov., holotype (MNHN-IM-2019-34864). **A** Dorsal girdle spicules (insert shows single dorsal spicule). **B** Bristle with spicules, marginal and ventral spicules. **C** Dorsal spicules, longer dorsal spicules clustered in groups and bristles. **D** Marginal spicules. **E** Ventral spicules in the anterior portion of hyponotum. **F** Spicules of precephalic tentacles.

Girdle broadly expanded anteriorly, uniformly brown or yellowish, dorsally covered with two kinds of spicules: short, single, densely arranged, smooth, and (mostly) mamillated spicules, L: 80–121 μm (mean = 106 μm, n = 7), W: 42–49 μm (mean = 45, n = 7), and longer, smooth and sharply-pointed spicules clustered in groups of a few, L: 241–313 μm (mean = 272 μm, n = 3), W: 28–35 μm (mean = 32 μm, n = 3) (Fig. 7A and C). Large bristles only present near the girdle margin and not prominent, beset with slender, smooth and sharply-pointed spicules, L: 240–280 μm (mean = 240 μm, n = 2), W: 30–40 μm (mean = 35 μm, n = 2), arranged in oblique series along axis (Fig. 7B). Marginal fringe composed of two kinds of spicules: straight, slightly striated (sometimes smooth), sharp-topped spicules, L: 129–152 μm (mean = 137 μm, n = 3), W: 25–30 μm (mean = 27 μm, n = 3), and second kind located closer to the ventral side: shorter and flattened, ovate spicules (Fig. 7D). Hyponotum covered in short, lanceolate-shaped, striated in the anterior half, sharply-pointed spicules; usually clustered in groups in the anterior half of the girdle, individually distributed in the posterior half, L: 50–82 μm (mean = 73, n = 6), W: 18–21 μm (mean = 20, n = 6) (Fig. 7E). Spicules of precephalic tentacles composed of two kinds of spicules: short, ovate-shaped, L: 47–64 μm (mean = 54, n = 8), W: 12–24 μm (mean = 19, n = 8), and long, lanceolate, smooth, which often cluster together and form a bristle similar to the dorsal ones, ca. 100 x 20 μm (Fig. 7F).

Radula of holotype small, ca. 4.5 mm in length in holotype, with 48 rows of teeth, of which 40 are of mature. Central tooth subtrapezoidal, with wide base and curved blade, first lateral tooth elongate, major lateral tooth with tricuspid head, denticles pointed, central denticle somewhat longer than others, outer denticle widest and shallowest notched (Fig. 6D).

Gills merobranchial, 11–13 ctenidia per side (12 and 13 on the left and right side, respectively, in holotype, 11 in paratype).

Paratype morphology mostly identical with holotype. Front margin of intermediate and tail valves stronger projected forward at jugal part, slits in head valve: 23, 30 incisions due to the irregular splitting. Smaller number of mamillated spicules on dorsum.

#### Diagnosis

Animal medium-sized, body broadly oval, valves subcarinated and moderately elevated, not beaked, tegmentum densely granulose. Tail valve roughly trapezoidal in shape, mucro terminal, raised and overhanging. Girdle expanded anteriorly, perinotum covered with two kinds of spicules: single, smooth, and (mostly) mamillated spicules, and longer, smooth and sharply-pointed spicules clustered in groups of a few; sparsely scattered large bristles beset with elongated slender spicules; hyponotum covered in short, lanceolate-shaped, striated in the anterior half, sharply-pointed spicules.

#### Etymology

The specific epithet *granulosa* is a feminine adjective formed from the Latin noun granulum = small grain, and the suffix -osa = “full of” highlighting the granular surface of the tegmentum characteristic of the new species.

#### Distribution

At present known only from its type locality, Papua New Guinea, Vitiaz Strait, western Pacific Ocean, at 05°59′ S, 147°39′ E, collected at 860–880 m depth.

#### Type material

Holotype (MNHN-IM-2019-34864) now partly disarticulated: mounts of shells (valves I, VII, VIII) and parts of girdle and radula mounted on three SEM stubs, respectively, and the vial with the specimen stored in 96% ethanol; one paratype (SMF 383080) now partly disarticulated: mounts of shells (valves I, II, VIII), and the vial with the specimen stored in 96% ethanol.

#### Differential Diagnosis

There are fifteen currently accepted species of *Placiphorella*, and six closely resemble *Placiphorella granulosa* sp. nov. by having number of slits in a head valve > 12 and granular tegmentum. It can be differentiated from:

*Placiphorella albitestae* Taki, 1954 by the more densely arranged, prominent, round granules, not thickened posterior margin of a head valve (vs. thickened to an obvious ridge in *P. albitestae*), and straight antemucronal slope (vs. concave in *P. albitestae*) (Taki 1954).

*Placiphorella atlantica* (A. E. Verrill & S. Smith, 1882) by terminal and overhanging mucro (vs. mucro subterminal in *P. atlantica*), longer spicules of the girdle (80-121 x 42-49 µm, smooth spicules, subtly mamillated in *P. granulosa* sp. nov. vs. 70-80 x 22-30 µm, striated with sharp beak on top in *P. atlantica*), striated ventral spicules (vs. smooth in *P. atlantica*) (Verrill 1882).

*Placiphorella laurae* Clark, 2019 by the trapezoidal shape of the tail valve (vs. broadly triangular, pointed in *P. laurae*), absence of radial sulci on head valve (vs. eight conspicuous sulci in *P. laurae*) (Clark 2019).

*Placiphorella methanophila* Vončina, 2024 by the shape of the VII valve (front margin almost straight and hind margin straight to weakly convex in *P. granulosa* sp. nov. vs. front margin widely angular and hind margin concave), roughly rectangular shape of the tail valve (vs. much wider, angular in *P. methanophila*), oblong and mamillated spicules of the perinotum (vs. single dorsal spicules sharply pointed in *P. methanophila*) (Senckenberg Ocean Species Alliance (SOSA) et al. 2024).

*Placiphorella isaotakii* Saito, Fujikura & Tsuchida, 2008 by the spicules of hyponotum (lanceolate-shaped, striated in the anterior half, sharply-pointed spicules up to 82 x 20 µm in *P. granulosa* sp. nov. vs. obtuse, smooth spicules up to 180 × 30 µm in *P. isaotakii*) (Saito et al. 2008).

*Placiphorella okutanii* Saito, Fujikura & Tsuchida, 2008 by the shorter and thicker spicules of perinotum (up to 121 x 49 μm vs. 150 x 30 µm in *P. okutanii*), the spicules of hyponotum (short, lanceolate-shaped, striated in the anterior half, sharply-pointed spicules in *P. granulosa* sp. nov. vs. obtusely pointed, smooth spicules in *P. okutanii*).

#### Notes

The obtained mitochondrial cytochrome oxidase subunit 1 (COI) gene sequence (GenBank accession number PX804842) was positively checked as belonging to *Placiphorella* against the GenBank database. The closest matches were sequences of *P. atlantica* with similarity of 94.48% (GU806116.1) or 94.39% (GU806076.1). The ranges of uncorrected genetic p-distances between *P. granulosa* sp. nov. and all other *Placiphorella* sequences available from GenBank in COI sequences ranged from 6% to 15.71% (Suppl. material 1).

## Discussion

The discovery of *Notoplax madagascariensis* sp. nov. and *Placiphorella granulosa* sp. nov. broadens our understanding of Indo-Pacific chiton diversity, a region renowned for its richness. *Placiphorella granulosa* represents the first record of the genus from Papua New Guinea, extending its previously northern Pacific distribution southward. Unverified *Placiphorella* records from New Zealand, attributed to *P. atlantica* in GenBank, likely represent additional undescribed species. *Notoplax madagascariensis* sp. nov. is the first *Notoplax* species documented from sublittoral depths off Madagascar. Its combination of *Notoplax* and *Leptoplax* diagnostic characters highlights the morphological overlap between these genera and the need for integrative taxonomic revision supported by molecular data. Broader phylogenetic sampling and continued exploration of under-surveyed regions such as Madagascar, Melanesia, and the Coral Triangle, will be crucial to resolving generic boundaries and uncovering the true extent of chiton diversity in the Indo-Pacific. The presence of several species of *Ferreiraella* near to Japan may indicate futher undiscovered diversity of this globally distributed group.

## Supplementary Material

XML Treatment for Ferreiraella populi

XML Treatment for Notoplax madagascariensis

XML Treatment for Placiphorella granulosa

1FE114A2-798D-510D-A631-AF4C0ECAF800 10.3897/BDJ.14.e180491.suppl1 Supplementary material 1 Genetic distances between *Placiphorella* species Data type genetic distance data Brief description Genetic distances between *Placiphorella* species collected from mitochondrial cytochrome oxidase subunit 1 (COI) partial gene pairwise comparisons. File: oo_1477737.xlsx https://binary.pensoft.net/file/1477737 Vončina
